# Have Cells Harboring the HIV Reservoir Been Immunoedited?

**DOI:** 10.3389/fimmu.2019.01842

**Published:** 2019-08-06

**Authors:** Szu-Han Huang, Chase D. McCann, Talia M. Mota, Chao Wang, Steven M. Lipkin, R. Brad Jones

**Affiliations:** ^1^Department of Medicine, Weill Cornell Medical College, New York, NY, United States; ^2^Program in Immunology and Microbial Pathogenesis, Weill Cornell Graduate School of Medical Sciences, New York, NY, United States

**Keywords:** HIV, cancer, latent reservoir, immunoediting, immunotherapy

## Abstract

Immunoediting is an important concept in oncology, delineating the mechanisms through which tumors are selected for resistance to immune-mediated elimination. The recent emergence of immunotherapies, such as checkpoint inhibitors, as pillars of cancer therapy has intensified interest in immunoediting as a constraint limiting the efficacy of these approaches. Immunoediting manifests at a number of levels for different cancers, for example through the establishment of immunosuppressive microenvironments within solid tumors. Of particular interest to the current review, selection also occurs at the cellular level; and recent studies have revealed novel mechanisms by which tumor cells acquire intrinsic resistance to immune recognition and elimination. While the selection of escape mutations in viral epitopes by HIV-specific T cells, which is a hallmark of chronic HIV infection, can be considered a form of immunoediting, few studies have considered the possibility that HIV-infected cells themselves may parallel tumors in having differential intrinsic susceptibilities to immune-mediated elimination. Such selection, on the level of an infected cell, may not play a significant role in untreated HIV, where infection is propagated by high levels of cell-free virus produced by cells that quickly succumb to viral cytopathicity. However, it may play an unappreciated role in individuals treated with effective antiretroviral therapy where viral replication is abrogated. In this context, an “HIV reservoir” persists, comprising long-lived infected cells which undergo extensive and dynamic clonal expansion. The ability of these cells to persist in infected individuals has generally been attributed to viral latency, thought to render them invisible to immune recognition, and/or to their compartmentalization in anatomical sites that are poorly accessible to immune effectors. Recent data from *ex vivo* studies have led us to propose that reservoir-harboring cells may additionally have been selected for intrinsic resistance to CD8^+^ T cells, limiting their elimination even in the context of antigen expression. Here, we draw on knowledge from tumor immunoediting to discuss potential mechanisms by which clones of HIV reservoir-harboring cells may resist elimination by CD8^+^ T cells. The establishment of such parallels may provide a premise for testing therapeutics designed to sensitize tumor cells to immune-mediated elimination as novel approaches aimed at curing HIV infection.

## Introduction

The cancer immunoediting hypothesis proposes that the immune system sculpts tumor immunogenicity even as it protects the host against the development of cancer. This occurs through a dynamic process consisting of three stages—elimination, equilibrium, and escape. Tumor elimination is the process through which the cancer immunosurveillance network is assembled, and drives the rapid elimination of tumor cells as they acquire somatic mutations. Equilibrium represents the period of immune-mediated clinical latency that follows the incomplete elimination of potentially cancerous cells, where the immune response and tumor engage in a cycle of tumor cell elimination, followed by selection and outgrowth of mutants escaped from immune pressure. The final stage involves the escape of tumor cells from immune control, resulting in the unrestrained outgrowth of the tumor. Cancer immunoediting was first reported in mouse models of cancer, where immunodeficient mice showed earlier and greater penetrance of carcinogen induction and spontaneous cancer development compared to wild-type mice (1–6). A substantial body of evidence now shows that this process is also prevalent in humans [reviewed in (1, 2, 7)]. Of particular importance, it has been shown that CD8^+^ T cells play an important role in cancer immunoediting, especially in cancers that acquire resistance to the adaptive immune response (8–10). In this Hypothesis and Theory article, we draw attention to the similarities between immunoediting in cancer and HIV, highlighting established and hypothetical parallels between tumor escape and the persistence of HIV-infected cells, and their potential implications on future applications of HIV cure strategies.

### Immunoediting in Cancer Evolution

Over the past several decades, there has been increased appreciation that adaptive and innate immunity can help sculpt the mutational landscape of cell lineages constituting tumors during cancer evolution and progression (3, 9–14), in some cases even before they are macroscopically detectable (15, 16). Observational studies have revealed that when either mice or patients are immunodeficient in adaptive immunity, incidence of certain types of cancer, including viral-induced cancers, increases (17–19). The overall process of how tumors are sculpted by adaptive and innate immune responses is referred to as cancer immunoediting (and less commonly, immune surveillance or immunoselection). While most studies of immunoediting have focused on T cell mediated immunoediting, a growing number of studies provide evidence highlighting the role that Natural Killer (NK) cells may play, particularly for tumor cells that have lost class I major histocompatibility complex (MHC) cell surface presentation (see below) (20–23).

The initial studies proposing the existence of immunoediting were largely drawn from studies of chemically induced mouse tumors in interleukin-2 receptor common subunit and VDJ recombinase (RAG) mutant mice that are immunodeficient in T cells, B cells, and NK cells (15, 16, 24). However, more recent studies evaluating the landscape of the specific mutations carried by individual tumors paired with the host patient HLA alleles provide additional evidence that tumor-specific changes in MHC-mediated antigen presentation affect tumor growth in humans (25, 26). All homeostatic nucleated human cells (except for certain testicular cell types that are immune-privileged) are decorated by class I MHC molecules on the cell surface membrane referred to as HLA. These molecules present proteasome degraded cytosolic 8–11 amino acid peptides to CD8^+^ cytotoxic T cells (CTLs) for recognition. Briefly, different dendritic cell populations (DCs) that encounter tumor cells can act as antigen presenting cells and present tumor antigens in the context of class II MHC [reviewed in (1, 2, 7)]. This cross-presentation by DCs expands and activates CD8^+^ cells, as well as CD4^+^ helper T cells that promote CD8^+^ cytotoxic T cell expansion.

Class I MHC HLA is encoded by three genes (HLA-A, -B, and -C) and is highly polymorphic. Different allelic combinations of HLA-A, -B, and -C, create significant diversity between individuals as to which antigens can be presented to CD8^+^ T cells. Typically, early in tumor development, cancer cells retain their HLA, and can be recognized and eliminated by immune cells if they present mutated host proteins (referred to as neoantigens). Additionally, cancer cells may over-express homeostatic antigens found in “normal” tissues (e.g., Mucin I (MUC1), or the HER2 growth factor receptor), that can have varying degrees of effect on central (thymic) tolerance. Recent studies (25, 26) show that, for human tumors paired with their patient host HLA from The Cancer Genome Atlas (TCGA), neoantigens with higher predicted HLA-neoantigen binding affinities, indicative of a higher likelihood of presentation to CD8^+^ T cells, were significantly more likely to experience mutations that decrease the HLA affinity of the targeted neoantigens. Additionally, these studies revealed that recurrent oncogenic mutations, such as KRAS or BRAF or IDH1 (collectively present on >35% of all solid tumors as well as many hematological tumors), have low predicted HLA-binding affinities. Thus, these paired tumor-host studies provide important new evidence that immunologically invisible human mutations are under an evolutionary selective pressure.

As mentioned above, cancer immunoediting is typically delineated into three stages: elimination, equilibrium and escape (9, 10, 20). Elimination is the first phase, whereby pre-malignant cells are killed by adaptive and innate immune cells patrolling normal tissues. This has been studied in mouse models, where both adaptive (T cell) (4, 8) and innate (NK cell) immunity (27–29) have important roles. For transformed cancer cells that evade elimination, perhaps starting even at the single-cell stage, cancer cell consortia form and enter the equilibrium stage. During the equilibrium stage, adaptive and innate immune cells kill some, but not all, tumor cells, leading to an evolutionary process whereby the epigenetic and somatic mutation landscape of tumor cells is sculpted. Consequently, although tumors may not appear to grow macroscopically, the “mutanome” of cancer clonal lineages that together comprise cancer consortia continuously evolve to promote immune escape. A common model to study this evolutionary process during equilibrium is colorectal cancer, as it is (a) often a relatively slow growing tumor, (b) a subset are hypermutators and have highly elevated mutations rates from DNA mismatch repair or DNA polymerase Delta/Eplison that can be tracked sequentially and (c) there are distinct histopathological stages that occur during its progression (e.g., normal colorectal epithelial crypts, aberrant crypt foci, dysplasia, carcinoma *in situ*, polyps and adenomas, frank carcinoma and metastases). Recent studies evaluating the landscape of tumor mutations during the evolution of colorectal cancers provide evidence that specific Single Nucleotide Variant (for example KRAS), small insertion/deletion (for example APC), and structural variants (e.g., TP53 loss), evolve, both as these lesions remain in equilibrium and also expand during progression (30–34).

Tumor cells that have acquired the pre-requisite mutations necessary to overcome immune pressure during equilibrium then enter the escape stage. The phenotypic changes required to reach this stage rely on a variety of factors, ranging from the geography of the tumor, to whether the cancer is liquid or solid. In solid tumors, an important step for immune escape is the development of an immunosuppressive microenvironment, known as the tumor microenvironment [TME, reviewed here (35, 36)]. This microenvironment is generally characterized by the secretion of immunosuppressive cytokines such as IL-10 and TGF-Beta [reviewed in (37–40)], nutrient scarcity imposed on immune effector cells by the ability of cancer cells to scavenge macronutrients from their environment (41), generation of a hypoxic environment that inhibits tumor infiltration and killing by T cells, B cells, and NK cells (42), and the promotion of a extracellular matrix that both enhances tumor cell growth while inhibiting immune cell penetration (43). While TMEs are not present in liquid cancers, similarities remain in how these cancerous cells escape from elimination, including: (1) the absence of a strong tumor antigen (44, 45), (2) the downregulation/loss of MHC-class I expression levels or co-stimulatory molecules (46, 47), (3) upregulation of exhaustion markers [e.g., CTLA-4, PD-L1, (45, 48)] (4) or the development of apoptosis resistant phenotypes due to increased expression of pro-survival proteins [e.g., BCL-2, MYC, STAT3, and 5, reviewed here (43)].

Interestingly, some of these characteristics that facilitate the escape of liquid cancers are similar to those seen in people living with HIV—Nef downregulation of MHC-I leads to low antigenicity of infected cells, and viral epitopes rapidly mutate in response to immune pressure, and escape immune recognition. Furthermore, our recent work has highlighted the inherent resistance of HIV-infected cells to immune-mediated elimination during suppressive anti-retroviral (ARV) therapy (49, 50). The following sections will highlight the potential mechanisms through which these phenotypes may arise, and discuss how the immunoediting of HIV-infected cells may occur.

### Treated vs. Untreated HIV Infection As Distinct Arenas for Immunoediting

**Figure 1:**
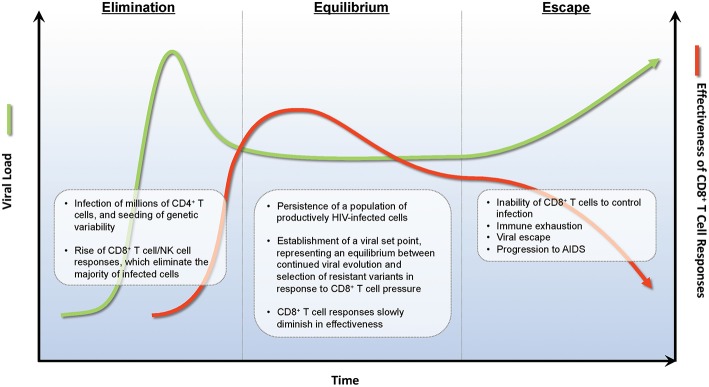
Immunoediting during natural infection. During the acute phase of infection, HIV rapidly expands infecting new target CD4^+^ T cells. Approximately 2 weeks post-infection, HIV-specific CD8^+^ T cell responses develop and eliminate many infected CD4^+^ T cells decreasing viral burden by ~1 × 10^2−3^ RNA copies/ml of plasma. A viral set point is reached when virus replication and CD8^+^ T cell elimination of infected cells reaches an equilibrium. During the equilibrium phase, ongoing rounds of viral replication and CD8^+^ T cell elimination provides evolutionary pressure to select for viral variants that are not recognized by CD8^+^ T cell responses. A combination of viral escape variants and CD8^+^ T cell exhaustion eventually leads to viral escape and progression to AIDS.

In the absence of ARV therapy, HIV infections are characterized by three stages—acute infection, chronic infection, and AIDS. Acute infection encompasses the first 4–8 weeks of infection, and is characterized by rapidly rising viral loads, often to >10^6^ copies/mL, and steep declines in the numbers of CD4^+^ T cells, both in circulation and in tissues (51). At ~6 weeks post-infection, robust HIV-specific CD8^+^ T cell responses develop that capably suppress HIV viremia to a set point that is typically 2–3 logs below peak (52, 53). While CD8^+^ T cells may control viral replication through a number of mechanisms (54–56), a key mode of action is the direct recognition and elimination of infected cells by CD8^+^ CTLs (57–60). This viral load set point is the primary characteristic of the second stage of HIV infection, known as the chronic phase, and represents the equilibrium between ongoing viral replication, viral immune evasion, and elimination of infected cells by the host immune response [reviewed in (61)]. Individuals with higher viral load set points progress more rapidly than individuals with lower set points to the final stage of HIV infection (62); where HIV eventually overcomes immune pressure in the large majority of individuals, leading to the onset of AIDS (Figure 1).

The current treatment for HIV is antiretroviral therapy (ART), which can durably suppress viremia to levels that are undetectable by clinical tests, and halt progression to AIDS for as long as treatment is maintained. However, using ultra-sensitive PCR methods, it has been shown that low-levels of virus production do persist in the majority of individuals (63), and are not reduced even if ART regimens are intensified (64, 65). Additionally, anywhere from 4 to 10% of people on ART may display levels of persistent viremia that are detectable by standard assays (50–500 copies/mL), even in the absence of drug resistance (66). Although there is strong evidence that ongoing cycles of viral replication do not occur during ART (67–69), uncertainty remains as to why HIV-specific CD8^+^ T cell responses do not seem to eliminate all infected cells that are producing viral particles. Lastly, upon ART cessation, viral loads rapidly rebound within a few weeks in the majority of individuals (70). This occurs despite the pre-existence of robust HIV-specific T cell responses which, though diminished in magnitude relative to untreated infection, are sustained at readily-detectable levels in most ART-suppressed individuals (71–74). While these studies seemingly highlight the limitations of CD8^+^ T cells in controlling and eliminating HIV-infections, multiple studies have unambiguously established the importance of CD8^+^ T cells in viral suppression (52, 57, 58, 75–78). Indeed, in non-human primate studies, CD8^+^ T cells are necessary for maintaining viral suppression of SIV during the course of both natural infections and ART (54, 79). These contrasting results raise important questions about why certain HIV-infected cells are efficiently eliminated by HIV-specific CD8^+^ T cell responses, while others persist, and may even continue generating viral particles during ART. While viral latency is known to play a critical role in HIV persistence, we will draw on insights from tumor immunoediting to propose additional cell-intrinsic mechanisms by which HIV reservoir-harboring cells may resist elimination by CD8^+^ T cells, and thus pose the question: have cells harboring the HIV reservoir been immunoedited?

## Immunoediting of the Virus During the Course of Untreated HIV Infections

A critical distinction in our discussion is between immunoediting of the virus during the course of untreated infections (which is a well-characterized phenomenon, although not typically branded as immunoediting), and the more novel idea that immunoediting may also occur on the level of reservoir-harboring cell physiology, particularly in the context of ART. The current section will focus on the former, which largely consists of the interplay between viral evolution and escape in response to CD8^+^ T cell pressure. While HIV infections are generally established by one to five “transmitter/founder” viruses (59, 80, 81), the high error rate of HIV reverse transcriptase (~1 point mutation per reverse transcription event, and a recombination frequency of ~2.8 crossovers) gives rise to a vast number of HIV “quasispecies,” each with varying degrees of replicative fitness (82–87). These mutations often incur a fitness penalty on the virus, as evidenced by the fairly homogenous makeup of viral sequences prior to CD8^+^ T cell pressure, despite the high mutation rate of HIV reverse transcriptase (88–91), and the presence of secondary compensatory mutations that arise in response. Despite these fitness costs, multiple lines of evidence have shown the importance of these mutations for viral replication, as they modify epitopes targeted by the host immune response and allow subdominant viral quasispecies to escape from immune recognition (75, 92–95). This mechanism of immune escape is well-documented in many longitudinal studies of HIV-infected individuals, where dominant CD8^+^ T cell responses can be matched to changes in the amino-acid sequence of targeted viral epitopes, leading to poor HLA presentation and the outgrowth of new HIV quasispecies (57, 96). Such escape on the level of viral epitope recognition is paralleled by the phenomenon of “antigen loss” in tumors—for example, the loss of MART-1 antigen in melanoma patients after adoptive transfer of MART-1 specific T cells (97, 98), or the loss of CD19 following CD19 targeted CAR T cell therapy for acute myeloid leukemia (99) [reviewed in (100)]. Thus, immunoediting on the level of viral sequence diversity has been well-established in HIV infection, and this “immune escape” is analogous to the phenomenon of tumor “antigen loss.” Antigen loss, however, is just one facet of tumor immunoediting, inspiring us to consider whether other mechanisms may also have parallels in HIV.

## Does Immunoediting At the Level of Infected Cells Occur in Individuals on aRT?

Immunoediting in untreated HIV infections involves the equilibrium between CD8^+^ T cell responses and HIV, followed by the eventual escape of HIV from CD8^+^ T cell killing in the majority of individuals. However, viral replication to a degree that allows for evolution does not occur during suppressive anti-retroviral therapy (67–69), preventing the development of new HIV escape mutations in response to CD8^+^ T cell mediated immune pressure. Instead, infected cells are thought to persist and evade the immune response during ART by hiding in a non-immunogenic quiescent, or latent, state. Infected cells that do not undergo this transition are largely eliminated: HIV DNA levels experience an 86% decline within the first year following ART initiation before stabilizing (101–103), while HIV RNA levels in the plasma drop precipitously over the first 7–10 days post-ART, with a half-life of 6 h, followed by a second phase of slower viral decay with a half-life of 14 days (104, 105). Infected cells that survive this selection and persist are remarkably stable, with a minimal half-life of at least 44 months as measured by total or intact HIV DNA, or by quantitative viral outgrowth assays (QVOAs) assessing the number of cells infected with replication competent proviruses (106–108). This suggests that the persistent reservoir would require at least 73 years to naturally decay in the majority of people living with HIV.

It is important to note that although HIV-specific CD8^+^ T cell responses decay sharply upon ARV initiation, in parallel with frequencies of HIV-infected cells, they are still readily detectable by *ex vivo* assays (ex. ELISPOT) in the large majority of individuals on long-term suppressive ART (71). The main paradigm for how infected cells persist during ART, despite the existence of CD8^+^ T cell responses, is that the reservoir “hides” from the immune system; this occurs primarily by maintaining a state of viral latency, but also through sequestration in anatomical sites that are poorly accessible to CD8^+^ T cells, such as lymph node follicles (109, 110). While these are indisputably important mechanisms of persistence, we propose that interactions between reservoir-harboring cells and CD8^+^ T cells are also likely to occur at some frequency in individuals on long-term ART (see Is Immune Selection Pressure Exerted on Infected Cell Clones During ART?, below), providing the potential for the shaping of the landscape of reservoir harboring cells in ways which may parallel tumor immunoediting.

Immunoediting is an evolutionary process, and thus will occur over time when the following three requirements are met: (i) reproduction, (ii) selective pressure, and (iii) heritable variation (14). The mechanisms by which these criteria are met in tumor cells are described above. Here, we make the case that these ingredients are also present in the persistent HIV reservoir, defined as follows: (i) reproduction—clonal expansion of HIV reservoir-harboring cells, (ii) selective pressure—ongoing immune recognition and clearance of certain reservoir-harboring cells, and (iii) heritable variation—genetic or epigenetic features of reservoir-harboring cells that confer differential susceptibility to immune recognition and clearance.

### Reproduction—Expansion of Clones of HIV-Infected Cells During ART

A major hallmark of cancer is the ability of cancer cells to promote continued expansion, even in a nutrient scarce environment, or lack of external stimuli. These hallmarks are a result of mutations in oncogenes (i.e., *MYC)*, which promote growth, or tumor suppressor genes (i.e., *p53*), which may inhibit cell division, repair DNA damage, or induce apoptosis if cellular functions become deregulated. In liquid cancers, the deregulation of c-*myc*—e.g., translocation from chromosome 8–14 in Burkitt's lymphoma (111)—generates abnormally high levels of MYC expression, resulting in enhanced cell cycle progression and cell growth (112). Conversely, p53 induces cell cycle arrest and apoptosis in the presence of cellular stress signals such as nutrient deprivation or DNA damage, and mutation of this gene allows cancer cells to continually proliferate under otherwise genotoxic conditions (113). Together, these gene mutations allow cancer cells to engage in constant clonal proliferation.

In contrast to cancer cells, the HIV-reservoir is thought to largely reside in long-lived resting memory CD4^+^ T cells, where the expansion and/or division of these cells are generally driven by either recognition of cognate antigen, or cytokine-induced homeostatic proliferation (114). Until recently, it was generally thought that an HIV-infected cell would be incapable of expanding in numbers, as cell division was thought to be inextricably linked to viral expression—which in turn, it was thought, would lead to death through viral cytopathic effects or immune-mediated elimination (102, 103). However, multiple studies have since demonstrated the ability of infected cells to proliferate *in vitro*. Hosmane et al. observed in QVOAs (115) that increasing numbers of cells producing replication competent viruses were found as CD4^+^ T cells were subjected to additional rounds of activation by mitogens (116), suggesting that cell activation and division are not intrinsically linked with reactivation of latent proviruses. Furthermore, a study by Bui et al. observed sustained levels of HIV RNA in a culture supernatant over 21 days, following activation with PMA/ionomycin, including sequences matched to replication competent viruses found in QVOAs. As these assays were performed in the presence of ARVs, these results demonstrate that production of replication competent viruses in reservoir-harboring cells does not necessarily lead to cell death (117).

The fact that HIV-infected CD4^+^ T cells can clonally expand *in vivo* was unambiguously established by the observation that 40–60% of all cells harboring proviruses had genomic integration sites that were identical to those of at least one other infected cell (118–121). Since HIV integrates into the genome without targeting specific sequences, it is extraordinarily improbable that the same integration site would occur independently in two separate cells, indicating instead that these cells clonally expanded from a common infected-cell ancestor. As the integration site loop amplification assay used to determine proviral integration sites (120) only amplifies a small portion of the 5′ and 3′ ends of the provirus, it was unclear whether these expanded clones contained intact proviruses, vs. the defective proviruses that make up the large majority of proviruses in individuals on long-term ART (ex. containing deletions, hypermutations, or other mutations that render them replication incompetent) (122, 123). It thus initially seemed that a simple potential explanation for how these cells could divide, without dying from cytopathic effects or immune elimination, was that they may contain defective proviruses—a subset of which are incapable of expressing virus or viral antigens (124). However, multiple studies have since provided evidence indicating that a subset of these clonally expanded populations can harbor intact, replication competent proviruses (125–128). These studies utilized QVOAs to isolate viral RNA from single viruses, and then assessed their clonality on the basis of viral sequences. It was inferred by phylogenetic and statistical approaches that these clonal proviruses almost certainly arose as the result of clonal expansion of the host cell, as opposed to the seeding of multiple infections by a single massive infection event.

More recently, a novel assay (matched integration site and proviral sequencing, MIP-seq) was developed to determine near full-length proviral sequences and the corresponding integration site simultaneously (129). This assay utilized a limiting dilution of proviral templates, followed by multiple displacement amplification to generate multiple copies of the proviral template and surrounding DNA, which could then be used for both full-length sequencing and integration site analysis. This approach definitively demonstrated that clonally expanded cells could indeed harbor intact proviruses (129). Moreover, the authors observed that these intact proviral sequences matched the sequences of viruses that had grown out in previous QVOAs from these same individuals. Thus, clonal expansion provides a mechanism through which the “replication with heritability” criterion of evolution may be fulfilled, accounting for the expansion of certain infected cell clones while others are eliminated.

### Is Immune Selection Pressure Exerted on Infected Cell Clones During ART?

Clonal expansion of HIV reservoir-harboring cells occurs in a setting where the overall size of the reservoir is relatively stable (106, 108). This implies that the death or elimination of some infected cells must occur on an ongoing basis, to counterbalance clonal expansion. A recent study examined this, by analyzing clonal composition of replication competent reservoir viruses (from viral outgrowth assays) longitudinally in 8 study participants. Wang *et al* found that while most of the clonal proviral populations were found at each time point throughout the course of the study, their proportional makeup of the total population differed at each time point (130). The authors observed a similar variation in the makeup of HIV clones found in the plasma of these participants, and concluded that populations of infected cell clones likely persist, but change in proportion relative to each other (“wax and wane”) over time. There are three possible and non-mutually exclusive explanations of these population dynamics: (i) stochastic effects—either random fluctuations of *in vivo* prevalence or in sampling, (ii) driven by the physiology of the CD4^+^ T cells themselves—ex. Expansion of a given clone driven by exposure to its cognate antigen, and iii) driven by fitness differences with respect to a selective pressure imposed on the infected cell.

In the oncology setting, a key determinant of whether or not a cancer cell clone will be subject to immune selection pressure is whether it possesses neoantigens that can be recognized as foreign by the immune system. In the case of an HIV reservoir-harboring clone, foreign antigens exist in the form of provirus-encoded viral genes. Moreover, these viral gene products are known to be immunogenic—in particular Gag, Pol, and Nef—and, in untreated infection, stimulate high magnitude T cell responses in the majority of infected individuals. In considering whether a reservoir-harboring clone is subject to immune selection, the key question is therefore the degree to which these gene products are expressed in an individual on ART.

In the large majority of individuals, cell-associated HIV RNA remains detectable at relatively low, but stable, levels in *ex vivo* CD4^+^ T cells even after years of suppressive ART (131). While the presence of viral RNA cannot be equated with protein expression, given that blocks to translation can exist at various levels, including splicing (132), and nuclear export (133), the transcriptional level data also are not counter-indicative of the possibility that antigen expression may occur at some level in individuals on ART. The direct assessment of HIV antigen expression in individuals on ART is limited by the much poorer sensitivity of protein vs. RNA detection assays, given the low frequency of infected cells. However, some studies have reported the detection of HIV proteins in *ex vivo* T cells from individuals on long-term ART (134). One way to infer whether ongoing interactions occur between the immune system and HIV in individuals on ART is to study the decay of HIV-specific immune responses in this context.

The maintenance of effector immune responses is dependent upon the presence of antigen (135–138). In addition to being a general tenet of immunology, this is supported by several lines of evidence in HIV. The first comes from the study of rare individuals who, without ongoing ARV therapy, exhibit extraordinary control over HIV infection as defined by undetectable plasma viremia by a single copy assay, extremely low to undetectable HIV DNA levels, and difficult to isolate replication-competent virus (139). These extremely low to absent levels of HIV were associated with the loss of HIV-specific antibody responses (sero-reversion), and with low to undetectable HIV-specific CD8^+^ or CD4^+^ IFN-γ responses in *ex vivo* PBMCs. *In vitro* stimulation did however result in the proliferation of HIV-specific T cells and subsequent antiviral activity, suggesting that cells had been present in a memory state. In contrast, while ART-treatment initially results in the decay of HIV-specific T cell responses with a half-life of 38.8 weeks for ~2 years (140), these then appear to stabilize, as HIV-specific T cell responses are readily detectable in *ex vivo* assays (ex. IFN-γ ELISPOT) in the large majority of individuals—even those who have been on treatment for over a decade (71). Similarly, while HIV-specific antibody responses wane upon initiation of therapy, ART-treated individuals do not sero-revert [with the exception of some individuals who initiate therapy very early (141)]. The second line of evidence for ongoing interactions between the immune system and HIV comes from the observation that the magnitudes of HIV-specific Ab responses correlate directly with frequencies of HIV-infected cells (HIV DNA) in individuals on long-term ART (142). Similarly, we have observed that T cell responses directed against the early HIV gene product Nef correlated directly with HIV DNA in this cohort (with Ab and T cell responses also correlating with each other) (71). While additional longitudinal studies are needed, these data are consistent with an ongoing interaction between HIV-infected cells and the immune system, including CD8^+^ T cells. Finally, it is interesting to note that the two individuals who achieved long-term remission of HIV through bone marrow transplantation—the “Berlin patient” and the “London patient”—also sero-reverted, and the London patient lost HIV-specific T cell responses (143, 144), though the ablation of the recipient immune systems does complicate the applicability of these cases to the current argument. Thus, while additional study is needed, we propose that the preponderance of evidence supports some level of ongoing interaction between the immune system and HIV-infected cells in individuals on long-term ART. If this occurs, it would satisfy the second criteria for the evolutionary process of immunoediting to occur—namely, selective pressure.

### How Might Reservoir-Harboring Clones Possess Heritable Variation in Susceptibility to Immune Clearance?

In the tumor immunoediting model, heritable variations generally arise during the cell replication cycle due to the failure of DNA mismatch repair enzymes to fix mutations, or when the cell fails to undergo apoptosis following a chromosomal break or translocation. Mutations that confer a selective advantage—through enhanced cell proliferation, resistance to apoptosis, and/or resistance to immune mediated elimination—are passed on to progeny cells, which will continue accumulating mutations that improve their survival or proliferative capabilities. Two major pathways that are mutated in many cancers, are those involved in MHC-I expression and BCL-2 overexpression, paralleling observations in reservoir harboring cells: the HIV protein Nef downregulates MHC-I expression, while Tat can upregulate BCL-2 expression. Here, we propose three potential sources of heterogeneity in the susceptibility of a given reservoir-harboring cell to immune-mediated elimination in ART-treated individuals: (i) virus intrinsic factors, (ii) host cell intrinsic factors (iii), and proviral integration sites.

#### Virus Intrinsic Sources of Heterogeneity in Susceptibility to CTL

Virus intrinsic mechanisms include variation in targeted epitopes that affect sensitivity to CD8^+^ T cell recognition, as discussed in section Immunoediting of the Virus During the Course of Untreated HIV Infections (above), as well as variable activity of viral immune evasion activity. As an example of the latter, it has been recently demonstrated that viruses reactivated from the reservoirs of ARV-treated individuals can vary greatly in their abilities to downregulate HLA-C through the actions of HIV-Nef (145). Of the three mechanisms of heterogeneity proposed here, these virus intrinsic mechanisms are the most well-established, and thus will not be a principle focus of this Hypothesis and Theory article.

#### Host Cell Intrinsic Sources of Heterogeneity in Susceptibility to CTL

With respect to host cell intrinsic mechanisms, it is known that various CD4^+^ T cell subsets display natural heterogeneity in their intrinsic susceptibility to CD8^+^ T cell-mediated killing. Effector and transitional memory CD4^+^ T cells are more susceptible to elimination than central memory CD4^+^ T cells (146), where the majority of the latent reservoir is thought to reside in (147). Another study observed that the CD4^+^ T cells of elite controllers were intrinsically more susceptible to CD8^+^ T cell mediated elimination than those from progressors, suggesting that CD4^+^ T cell sensitivity to killing may play a role in disease outcomes (148). Although the mechanisms underlying this heterogeneity within the CD4^+^ compartment are not well-understood, multiple mechanisms of resistance are known in other cell types. CTL protect themselves from this killing process by inactivating perforin through Cathepsin B or CD107a expression (149, 150). Similarly, macrophages and dendritic cells avoid being killed by expressing serine protease inhibitors that degrade granzyme B (151–154). BCL-2 can also confer resistance to CTL further downstream in both the perforin/granzyme B and FasL/Fas pathways by sequestering Bid, thus preventing mitochondrial membrane permeabilization by tBid (155, 156). In recent work, we have identified one mechanism by which HIV reservoir harboring cells are disproportionately resistant to CTL killing: through the over-expression of the prosurvival factor BCL-2 (50). Interestingly, previous studies have also described a disparate role for BCL-2 in the survival of reservoir-harboring cells through prevention of apoptosis mediated through Casp8p41, an HIV-protease cleavage product of procaspase-8 (157–159). While this is a fairly nascent area of research, barring the null hypothesis—which is that all CD4^+^ T cells are precisely equal in their susceptible to killing—it stands to reason that any heritable variation in susceptibility to CTL will influence which infected cells survive to form the persistent reservoir, and thus the subsequent sensitivity of the reservoir to immune-mediated clearance.

#### Proviral Integration as a Potential Source of Heterogeneity in Susceptibility to CTL

Likely the most provocative of our proposed sources of heterogeneity in sensitivity to CTL is the potential role of proviral integration sites. As a retrovirus, a defining step in the lifecycle of HIV is integration of the proviral DNA into the host genomic DNA. After reverse transcription generates a double stranded cDNA of the viral RNA, the reverse transcription product is shuttled into the nucleus via the nuclear pore complex as part of a pre-integration complex (PIC). Once inside the nucleus, Integrase (IN) resects 2 nucleotides from both 3′ ends of the viral DNA molecule, binds the target genomic DNA, then makes a 5-nucleotide staggered cut in the host DNA allowing for transfer of the viral DNA onto the host genome where host enzymes, DNA polymerase and ligase, fill in the gaps and irreversibly ligate the two DNA strands together—now designated the HIV provirus [reviewed in (160)]. While HIV integration occurs across the human genome, the chromosomal location of integration is not completely random. *In vitro* studies have shown that HIV preferentially integrates into actively transcribed genes, gene-rich regions, intronic regions, and largely avoids promoter regions (161). Preferences for these sites are largely mediated by cellular cofactors that bind IN and possess chaperone-like and chromatin tethering activity, most notably, the transcriptional activator LEDGF/p75 (162, 163). While LEDGF/p75 plays an important role in guiding chromosomal integration, it is not a necessary factor as loss of LEDGF/p75 showed no decrease in the overall frequency of HIV integration, but instead resulted in an altered proviral landscape (164).

*In vivo* studies of patients on long-term ART corroborated *in vitro* findings with a preference for HIV integration into transcriptionally active genes, and principally within introns (165–167). *In vivo* studies have also identified large, clonally expanded populations of HIV-infected cells with integration sites within genes controlling cell growth and division (120, 121). Of note, multiple patients have been identified with integrations in the BACH2, MKL2, and STAT5B genes. *In vitro* infections of primary cells demonstrated integrations throughout BACH2 and MKL2 with equal distribution of chromosomal orientation. However, large clonally expanded proviral sequences from patients on long-term ART were all in the same orientation as gene transcription and found only within a specific subset of introns (121). While the exact mechanism of survival is not completely understood, it is believed that BACH2 and MKL2 gene expression may be driven by the HIV LTR promoter [reviewed in (161)]. The existence of these clonal integrations within genes associated with cell growth in patients on long-term ART strongly suggests a role in maintaining the persistent reservoir through the induction of clonal expansion. However, to our knowledge, there are currently no studies extensively evaluating the impact that the site of HIV integration may have on maintaining the persistent reservoir by providing a mechanism of resistance to immune recognition and clearance.

Our hypothesis that HIV proviral integration sites may alter susceptibility of target cells to CTL recognition and elimination was inspired by findings related to immunoediting in cancer. Immunotherapies have recently achieved remarkable success in the treatment of certain types of cancer, but exhibit variability in responses across patients (168). A recent study of patients undergoing anti-PD-1 therapy (pembrolizumab) for metastatic melanoma who experienced cancer relapse after tumor regression, found that a majority of relapsing cancer cells contained somatic mutations in genes associated with interferon receptor signaling (JAK1 and JAK2) or antigen presentation (B2M) (169). These cancer cells were therefore less responsive to IFN-γ or had reduced MHC-I surface expression, leading to escape from immune-mediated control. Additionally, a number of groups have employed high-throughput CRISPR screens to identify genes controlling susceptibility/resistance to immune clearance (170, 171). Using a large-scale CRISPR screen of a melanoma cell line, Patel et al. found that disruptions in antigen processing/presentation and IFN-γ signaling pathways resulted in decreased CD8^+^ T cell effector functions (170). The top hits identified in the CRISPR screen were compared back to the TCGA database where it was demonstrated that identified mutations in these genes naturally occur in human cancers. Thus, the acquisition of resistance to CTLs by tumors can underlie poor responses to immunotherapy.

Integration of the HIV genome into cellular genes has parallels with cancer-induced mutations or CRISPR-mediated disruptions, leading us to posit that HIV integration into genes essential for immune recognition and signaling could reduce CD8^+^ T cell killing of those cells, thereby resulting in an immunoedited subset of survivor cells enriched for integrations in those genes. A few important differences, however, exist between CRISPR-mediated gene disruptions and those caused by HIV proviral integration. First, CRISPR gRNA libraries are developed to specifically target exonic regions, resulting in loss-of-function mutations. As discussed previously, the vast majority (93–96%) of HIV integrations occur within introns (165–167). The impact of a ~9 kb intronic insertion containing an LTR promoter, or of a truncated defective provirus, depends upon a number of factors, and could plausibly increase, decrease, or not at all impact gene expression and/or protein function. Second, HIV only integrates into a single locus of a given gene whereas CRISPR-mediated cleavages typically disrupt both alleles of the target gene. Therefore, HIV integration into a single allele may not impact overall protein function. However, a number of genes exhibit haplo-insufficiency, whereby a single copy of the gene product is not sufficient to support normal gene function, and thus disruption in a single allele may disrupt normal gene function; either wholly or on a nuanced level [reviewed in (172)].

**Figure 2:**
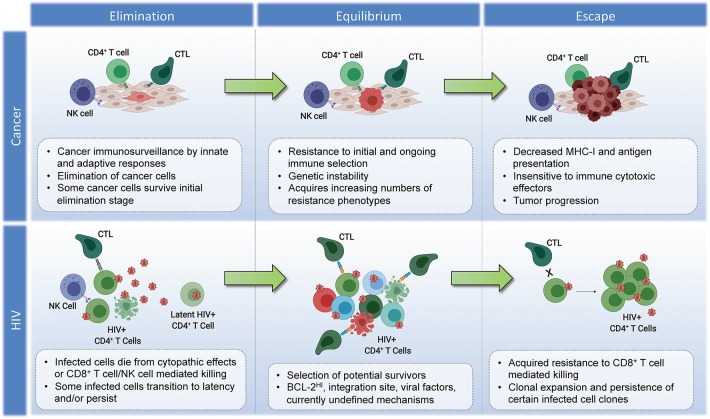
Parallels in immunoediting: comparing cancer and HIV during suppressive ART. Cancer–Elimination: Innate and adaptive immune cells work together to destroy developing tumors before they become clinically apparent, which play critical roles in cancer immunosurveillance. Highly antigenic tumor cells are recognized and eliminated by increased antigen presentation and IFN-γ, NKG2D, TNF, IL-12, TRAIL, Perforin, and Granzymes. Equilibrium: Tumor cells that survive elimination may enter the equilibrium phase. T cells, IL-12, and IFN-γ work in tandem to maintain the tumor cells in a state of functional dormancy. Tumor cells are in a state of genetic instability, and acquire an ever-increasing number of mutations to resist immune pressure. Escape: Tumor cells surviving the equilibrium phase of the cancer immunoediting process enter the escape phase, where tumor growth is no longer blocked by immunity. Tumor cell evasion generally occurs in cases with poor antigen presentation, and increased tumor-derived immunosuppressive cytokines, ligands, and inhibitors of T cell responses (see section Immunoediting in Cancer Evolution). Tumor cells escaped from immune pressure can grow unchecked, resulting in clinically apparent disease progression. HIV–Elimination: Seeding of millions of infected cells, each with a unique viral and host cell signature. The majority of infected cells die from viral cytopathicity or immune-mediated elimination following ART initiation. Some infected cells persist. Equilibrium: low-level/episodic antigen presentation allows for ongoing selection of infected cells. Some infected cell clones are eliminated, while others persist and expand. The overall number of infected cells remains stable. Escape: Expansion of infected cell clones with characteristics that enhance their resistance to immune recognition and/or elimination.

While it is possible that the site of HIV integration may impact the susceptibility of an individual cell to recognition and/or elimination by CD8^+^ T cells—thereby providing a means of immunoediting—further research is needed to determine if this is indeed a genuine HIV-induced survival mechanism. There are quite daunting challenges involved in testing this hypothesis: (i) The fact that most proviral integrations in ARV-treated individuals are associated with defective proviruses—many of which are non-antigenic—comprise a source of “noise,” since only the minority of antigen-expression competent proviruses would potentially be subject to immune selection. Thus, bulk integration site analysis would be expected to miss any selection for integration sites that affect immune susceptibility. (ii) There is extensive complexity inherent in both the vast landscape of potential unique integration sites across the genome, and the divergent impacts that any potential integration could have in terms of gain/loss of function, or more exotic effects such as the generation of novel chimeric proteins (173). This will likely make it much more difficult to discern patterns than simple CRISPR loss of function mutations. (iii) Any selection on the level of integration sites would occur on the backdrop of differential susceptibility to CTL on virus- or host-cell intrinsic levels. As a simple example, an infected cell with an integrated provirus that contains escape mutations to autologous CD8^+^ T cell responses would be exempt from any putative selection on the level of integration sites, and thus would confound analysis if not accounted for. Despite these challenges, the question of whether or not integration sites affect immune susceptibility may be addressable if one were to effectively harness novel approaches to obtaining integration sites in conjunction with whole provirus sequences; and to apply sophisticated analytical approaches. Inspiration for how this might be approached can be drawn from the study of cancer immunotherapy resistance—ex. the TIDE (Tumor Immune Dysfunction and Exclusion) computational framework, which draws on transcriptomic signatures from 33,000 samples taken across 189 studies to predict immune checkpoint blockade responses and derive insights into immunotherapy resistance mechanisms (174). While the outcomes of such efforts in the setting of HIV may indeed be to find that integration sites have no bearing on susceptibility to CTL, the alternative result would both have important implications for efforts to cure HIV infection, and would comprise a potential source of fundamental immunological insights—with the potential to cross-fertilize our understanding of cancer immunoediting. The proposed parallels between the immunoediting of tumors and the persistent HIV reservoir are summarized in Figure 2.

## Implications of a Persistent Reservoir That Has Been Immunoedited

The potential ongoing selection of certain infected cell populations *in vivo* during suppressive ART has many implications for current cure approaches, and may help explain the differential outcomes of these strategies *in vitro* vs. *in vivo*. One particularly prevalent approach, termed “kick-and-kill,” combines latency reversing agents to initiate viral transcription, ARVs to prevent viral spread, and effectors to eliminate reactivated virus-harboring cells. While applications of kick-and-kill initially had shown great promise in primary cell models of latency (175), these approaches have, thus far, not measurably reduced the latent reservoir in multiple clinical trials (176–182). Multiple studies have also attempted to apply kick-and-kill approaches in further *in vitro* or *ex vivo* models, but have not definitively shown reductions in the natural, replication competent reservoir (49, 179, 183, 184), suggesting that there are intrinsic differences in susceptibility to CD8^+^ T cell killing between natural and model reservoirs.

One possibility is that the remaining infected cells comprising the latent reservoir may be adapted to survive the host immune response, as our group has provided evidence that cells harboring the latent reservoir may be intrinsically resistant to CD8^+^ T cell killing (49). We combined maximal T cell activating agents, such as stimulation using anti-CD3/CD28 antibodies or PMA/ionomycin, with autologous HIV-specific CD8^+^ T cell clones targeting non-escaped epitopes, and still failed to detect decreases in the number of replication competent proviruses by QVOA. We then harvested the replication competent proviruses that grew out in the QVOA, which are individual clonal lineages due to the limiting dilutions utilized in QVOAs, and super-infected activated CD4^+^ T cells from the same donor. We then co-cultured these newly infected cells to the same HIV-specific CD8^+^ T cell clones as before, and observed elimination of nearly all the infected cells. These contrasting results of efficient CD8^+^ T cell elimination of infected cells during productive infections, but inability in eliminating latently infected cells, suggests that there likely are host-cell associated factors that impact survival of latently infected cells.

Drawing from the tumor immunoediting literature, we identified overexpression of the pro-survival protein BCL-2 as one potential mechanism of resistance—which can act to antagonize perforin/granzyme killing by sequestering truncated BH3-only domain members of the BCL-2 family (185). Using cells from individuals on long-term ART, we observed that reactivated HIV reservoir-harboring cells from *ex vivo* CD4^+^ T cells over-expressed BCL-2 relative to uninfected cells (50). In contrast, we did not observe over-expression in *ex vivo* HIV infected cells from ART-naïve individuals—suggesting that this was a unique feature of long-term reservoirs. The addition of the BCL-2 antagonist ABT-199 to combinations of HIV-specific CD8^+^ T cells and latency reversal agents resulted in partial eliminations of *ex vivo* reservoirs from ARV-treated individuals. We propose that these results comprise proof-of-principle for the idea that reservoir-harboring cells may be selected for resistance to CD8^+^ T cells, but would suggest that BCL-2 over-expression may be just one of many mechanisms yet to be discovered.

Other long-term implications of the potential immunoediting of persistently HIV-infected cells during suppressive ART are unclear. In a paired submission to this same issue, we discuss in detail a model of virus- and host-coordinated immunoediting of a retrovirus that causes cancer: adult T cell leukemia/lymphoma arises in ~5% of individuals living with the Human T cell leukemia virus type 1 (HTLV-1), although the development of malignancy can take 40–50 years (186, 187). The HTLV-1 specific immune response acts in concert with cancer immunosurveillance, driving the proliferation of immortalized immune-evading infected clones with identical integration sites that may acquire properties through years of equilibrium that can drive malignancy. Virus- and host-coordinated immunoediting sculpts the selection of a single clonal population to become malignant after decades of latency and clonal expansion (188). Although HIV does not persist through the classical escape phase and is not known to cause T cell malignancy, the immunoediting of HIV-infected cells that persist through ART may drive the selection of clonal populations of cells arising from a single integration site, which persist indefinitely. We argue that this persistence may represent the escape phase for people living with HIV on ART, particularly for clonally expanded cells harboring replication competent HIV that remain refractory to immunosurveillance and survive for years. The fate of these cells remains unknown, and understanding the mechanisms of their survival will ultimately inform their capacity to be purged.

## Summary

The recent revolution in cancer immunotherapy has underscored the potential for the human immune system to combat tumors, and shone a spotlight on the diverse mechanisms by which cancers can acquire cell-intrinsic immune resistance. Using sophisticated Omics approaches, and cutting-edge technologies, it has been revealed that both the genetic and epigenetic features of a given tumor cell can influence its intrinsic sensitivity to immune recognition and elimination. This variation serves as the basis for an evolutionary process known as clonal selection, which leads to the escape of tumors that have been immunoedited. Some mechanisms of immunoediting may be therapeutically targetable—e.g., IFN-γ treatment to augment antigen processing and presentation, or PD-L1 blockade for tumors that overexpress this co-inhibitory ligand (189). In contrast, the field of HIV persistence has generally not considered the idea that reservoir-harboring cells themselves may differ intrinsically in their susceptibilities to CTL, focusing instead on the roles of virus expression/latency, and on aspects of CTL functionality. Here, we have attempted to build a case for the potential role of cell-intrinsic immunoediting in the persistence of the HIV reservoir; including preliminary evidence supporting this model, suggested mechanisms for how this may arise, and a discussion of how this theory can be further evaluated. In moving forward, we propose drawing on the concepts, technologies, and methodologies that have been developed to study tumor clonal section and immunoediting to accelerate progress toward understanding the nature of HIV persistence, and how this may be overcome to cure infection.

## Data Availability

No datasets were generated or analyzed for this study.

## Author Contributions

All authors listed have made a substantial, direct and intellectual contribution to the work, and approved it for publication.

### Conflict of Interest Statement

RJ declares that he is a member of the AbbVie Inc Scientific Advisory Board, and has received payment for this role. The remaining authors declare that the research was conducted in the absence of any commercial or financial relationships that could be construed as a potential conflict of interest.

## Abbreviations

CTL: Cytotoxic T lymphocyte
HIV: Human Immunodeficiency Virus
ARV: Antiretrovirals
ART: Antiretroviral therapy
QVOA: Quantitative viral outgrowth assay
PCR: Polymerase chain reaction
TCGA: The Cancer Genome Atlas
TME: tumor microenvironment.
